# Making the PI and PID Controller Tuning Inspired by Ziegler and Nichols Precise and Reliable

**DOI:** 10.3390/s21186157

**Published:** 2021-09-14

**Authors:** Mikulas Huba, Stefan Chamraz, Pavol Bistak, Damir Vrancic

**Affiliations:** 1 Institute of Automotive Mechatronics, Faculty of Electrical Engineering and Information Technology, Slovak University of Technology in Bratislava, 812 19 Bratislava, Slovakia; stefan.chamraz@stuba.sk (S.C.); pavol.bistak@stuba.sk (P.B.); 2 Department of Systems and Control, Jožef Stefan Institute, Jamova Cesta 39, 1000 Ljubljana, Slovenia; damir.vrancic@ijs.si

**Keywords:** filtration, multiple real dominant pole method, PI and PID control, derivative action, anti-windup, digitization

## Abstract

This paper deals with the design of a DC motor speed control implemented by an embedded controller. The design is simple and brings some important changes to the traditional Ziegler–Nichols tuning. The design also includes a novel anti-windup implementation of the controller and an integrated noise-reduction filter design. The proposed tuning method considers all important aspects of the control, such as pre-processing of the measured signals and filtering (to attenuate the measurement noise), time delays of the process, modeling and identification of the process, and constraints on the control signal. Three important aspects of designing PI and PID controllers for processes with noisy output on Arduino-type embedded computers are considered. First, it deals with the integrated design of the input filter and the controller parameters, since both are interdependent. Secondly, the method of setting the controllers from step responses by Ziegler and Nichols is modified for the case of digital signal processing (without drawing the tangent), while it recommends the suitability of its modification in terms of the use of both integral and static models. Third, the most suitable anti-windup solution for the given controller structure is proposed. In summary, the paper shows that an appropriate design of the embedded controller can achieve excellent closed-loop performance even in a noisy process environment with limited control signals.

## 1. Introduction

The introduction of simple single-chip microcomputers has revolutionized process control, which no longer depends on hardware alone. The combination of multiple functionalities in one device makes it possible to bypass the traditional division of the control loop into sensor, controller and actuator. At the same time, the integrated design allows for simplified and accelerated solutions, resulting in increased control performance and robustness.

The concept of PI and PID controllers has been developed in several stages. After their discovery more than a hundred years ago, PI controllers were considered a highly valued commodity, forming a control loop together with sensors and actuators. In the 1930s, a derivative component (called Pre-Act) was added to the PI controllers. The terms proportional-integral or proportional-integral-derivative, from which the abbreviations PI and PID are derived, came later in history. The modular industrial control loops, originally based on pneumatic, hydraulic and electromechanical solutions, changed over time with the advent of transistors, analog integrated circuits and digital technology. The introduction of cheap and powerful embedded computers made it possible to process control algorithms and sensor signals simultaneously. The benefits of integrated design of control algorithms and process signal filtering are so great that they can change the traditional clichés found in control engineering textbooks. The assertion “for noisy systems, it is not appropriate to use controllers with derivative action” [1] is no longer valid and should be changed to “for noisy systems, controllers with derivative action should be used in conjunction with appropriate filtration” [2].

Since its publication, the method for tuning controller parameters by Ziegler and Nichols [3] has been one of the most popular and widely cited, and has inspired many subsequent improvements [4,5,6,7,8,9,10,11]. Originally, the method focused on the best possible suppression of disturbances, to which was also adapted the choice of more “aggressive” settings of controllers, leading mainly to oscillating transients with the so-called “quarter amplitude damping”. With the advent of digital computers, the tuning methods were first discretized by introducing the discrete-time transfer functions of the controllers [12], to allow using with longer sampling periods. Later, the numerically sensitive computation of the tangent drawn through the inflection point of the measured step responses was replaced by simpler approximations of the initial segment of the setpoint step responses [13], generalizing the method to higher-order models as well. In this paper, we will focus on digitization yielding two types of linear models obtained from the step responses. The method is further refined to ensure fastest possible transients without overshoot and to allow generalization to higher-order approximations [14].

The systematic design of controllers for noisy systems requires several crucial steps. The first is to appropriately quantify the performance achieved when evaluating transients. The use of traditional statistical tools does not seem appropriate here, since relevant conclusions can only be drawn after a very lengthy investigation (large amount of processed data). On the other hand, the controller must guarantee the required control performance for all considered control processes without requiring a high number of repetitions. Moreover, the noise of the process measurements (sometimes with considerable amplitudes) must be taken into account at the initial stage of the control design, since high peaks in the noise signals could affect the lifetime of the installed electronic devices. The appropriate criteria for evaluating the performance of the achieved process and control transients are based on the early work of Feldbaum [15] in the field of time-optimal control. In [15], it was shown that *n* control intervals at the process input are generally required for an *n*th-order system to make a time-optimal transition from one steady state to another, with the control signal alternately changing value between two control signal boundaries. Although Feldbaum’s theorem was later forgotten with the advent of linear PID controllers (several alternative approaches to constrained control of time-delayed systems may e.g., be found in [16,17]), it is noted that the theorem is becoming increasingly important as the dynamics of transients increase. This fact was already highlighted in the context of PID control [18] and led to the concept of dynamic classes of control [14,18,19]. Indeed, when designing PID controllers, it is necessary to define the dynamic class of control for which it is proposed.

In the case of a PI controller for a stable first-order system, the ideal course of the control signal following the step of the reference variable to the new constant value may be monotonic, but also with one pulse (1P), i.e., consisting of two monotonic sections separated by an extreme. Instead of a single extreme point, the monotonically increasing/decreasing segments can be separated by a section at a control signal saturation limit. With unstable systems, only the second option is possible. However, the same input and output shapes can be achieved using a PID controller. When designing PID controllers for second-order systems, a third possibility will be added, when the optimal course of the control signal following a setpoint or input disturbance step will consist of two impulses (2P), i.e., three monotonically increasing or decreasing sections separated by two extremes or sections at saturation limits. This means that several types of controllers from the same dynamic class can be designed for the same system, but sometimes several controllers of the same type from different dynamic classes. Examples of controller tuning for second-order systems can be found in [13,20,21,22,23,24,25,26]. A broader collection of different approaches gives [27]. Quantitative criteria for evaluating deviations from ideal shapes of transients of process input and output signals have been developed based on deviations from monotonicity [14,28]. In the context of this work, we restrict the design of PI and PID controllers to obtaining a 1P control signal after setpoint and input disturbance steps.

In [3], the relationships for setting the regulators were determined empirically for a wider batch of processes. Given that PID controllers at the time represented a dominant part of the existing supply of industrial regulators, such a procedure could have been natural. However, today’s offer, especially if we take into account practically universal programmable embedded controllers, already allows a closer adaptation of the optimal design to a specific type of process. From this point of view, we do not consider the efforts of some newer modifications of the method for universally valid settings [8,9] to be justified. The given trend of narrowing the processes for which the design was optimized can be seen already in the work [12], in which the IPDT system model plays an important role. Given that the IPDT model did not allow a simple explanation of several aspects of the original work of Ziegler and Nichols, in our contributions we also focused on the analysis of other type processes, such as integrator + time constant delay [6,7], double integrator + dead time [13,20,26], or (following the example of the work [29]) approximations with higher-order time constants and transport delay [14]. Instead of numerically ill-conditioned determination of the inflection point and problems with evaluation of steady states, we also preferred the digitization of the method based on the best possible approximation of the introductory part of the measured step response by FOTD and IPDT models. From the closed-loop transients, we expected to reach the highest possible speed under the given control constraints, with the minimal deviations from ideal shapes of piecewise monotonic responses [13,20]. The paper, which is an amalgamation of several publications on the subject (see, e.g., [2,30,31,32,33,34,35,36,37,38,39,40,41,42,43,44,45,46,47,48]) provides a refinement of the original step-response-based method of Ziegler and Nichols in terms of the nearly time-optimal responses to step setpoint and input disturbances changes in systems with limited control action also under the influence of measurement noise. It is written pragmatically with the aim of making the subject accessible to the widest possible audience. It is organized as follows: After the basics of system modeling based on approximations of their process response curves by IPDT and FOTD models in Section 2, Section 3 discusses the optimal integrated design of PI and PID controllers with input filters and control signal limitations. Section 4 gives criteria for evaluating the optimality of the closed-loop transients achieved. Section 5 describes an experiment with an electromechanical system. The obtained results are evaluated in Section 6 and summarized in the Conclusions.

## 2. Possible Approximations of Stable Setpoint Step Responses

**Figure 1:**
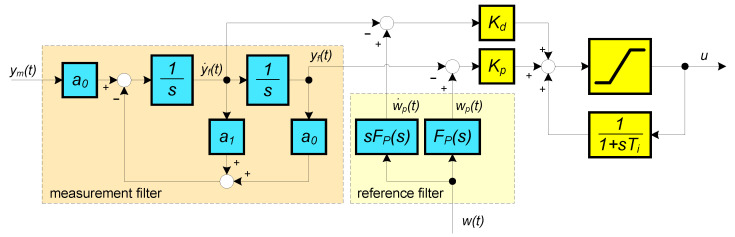
A 2nd-order binomial filter of the measured variable $y_{m}\left(t\right)$ proceeding an anti-windup implementation of PID controller with proportional and derivative gains $K_{p}$, $K_{d}$, with the integral time constant $T_{i}$, with the reference setpoint w(t) and a prefilter $F_{p}\left(s\right)$.

The measured process output signal must be filtered before it is further used in the PID controller (see Figure 1) to remove unwanted noise that could reduce the lifespan of the motor and its driver circuitry. Therefore, in addition to the filter, voltage limiters (implemented by diodes) are added to the input of the circuit.

### 2.1. Input Filter Design

To obtain a filtered velocity signal from the motor ($y_{f}\left(t\right)$) or even its phase vector components $y_{f},{\dot{y}}_{f},{\ddot{y}}_{f},\cdots y_{f}^{n-1}$ from raw velocity measurements ($y_{m}\left(t\right)$), a binomial low-pass filter $Q_{n}\left(s\right),n>1$ is used, which is given by the transfer function

(1) $$\begin{array}{c} Q_{n}\left(s\right)=\frac{Y(s)}{Y_{m}\left(s\right)}=\frac{1}{{\left(T_{f}s+1\right)}^{n}}=\frac{a_{0}}{s^{n}+a_{n-1}s^{n-1}+\cdots +a_{1}s+a_{0}};\\ a_{k}=\frac{n!}{\left(n-k\right)!\;k!\;T_{f}^{n-k}};\;n=1,\;2,\cdots \;;\;k=0,1,2,\cdots ,n;\;n!=n\left(n-1\right)\left(n-2\right)\;\cdots \;3*2*1 \end{array}$$

Please note that $Y_{m}\left(s\right)$ is the Laplace representation of the raw signal $y_{m}\left(t\right)$. The coefficients $a_{k}$ are calculated by the Pascal’s triangle. Please note that for a quasi-continuous-time filter implementation with a sampling period $T_{s}$, $T_{f}$ must satisfy the following condition:

(2) $$T_{s}<<T_{f}$$

Then, the integration block labeled 1/s in Figure 1 can be replaced by a summation performed by a control program implemented in the Arduino environment. The equivalent time delay of the filter can be represented by its average residence time (ART) [1]

(3) $$T_{ART}=nT_{f}$$

Please note that as the order of the filter *n* increases, the value of $T_{f}$ satisfying the condition $T_{ART}=const$ decreases down to the sampling period $T_{s}$, which is the fundamental constraint for the choice of *n*. In the rest of the paper, the filter parameters are calculated according to the process and sensor analysis. To simplify the design of the controller, the identification of the process will include the built-in filter.

### 2.2. Measuring Input-Output Steady-State Characteristic

When designing controllers for stable systems, it is always important to obtain the steady-state input-output characteristics (IOSSCH) of the process and determine the limits of the system variables. These limits are considered in the identification phase and in the evaluation of the efficiency of the control system. Based on the limits, an appropriate anti-windup protection is also applied. It should be noted that the overall control design also depends on the signal-to-noise ratio.

**Figure 2:**
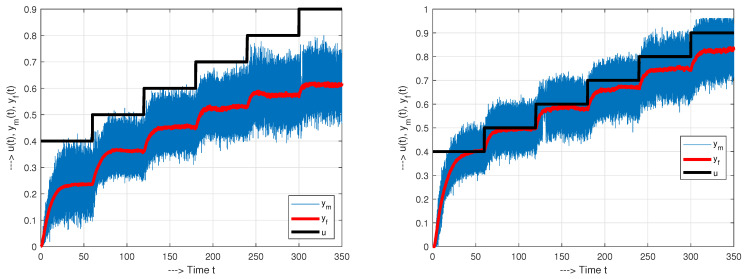
IOSSCHs of a mechatronic system with a measured output $y_{m}$, a filtered output $y_{f}$ and a process input pulse-width *u* measured before and after maintenance (bearing lubrication).

In the identification phase, the dynamic characteristics of the process are determined. The accuracy of the model is usually evaluated using various quantitative criteria, such as the sum of the squares of the difference between the output of the real system and its model. However, the obtained model is best verified by evaluating the closed-loop transients obtained with the controller designed according to the determined model. As mentioned earlier, the main objective of measuring the IOSSCH of the process is to obtain limit values for the process input and output. The output of the controller is then limited according to the measured process limits. The process limits can be determined in the open loop configuration. If the process is too slow, the closed-loop configuration can be used with a stable control. When the process settles, the pairs of values of process input and process output should be recorded. If the IOSS curve $y_{f}=F\left(u\right)$ is not linear, it is useful to use the inverse characteristic at the controller output to linearize it. Two examples of measuring the IOSSCH together with measured ($y_{m}$) and filtered ($y_{f}$) output signals are shown in Figure 2.

The use of embedded controllers is often associated with the use of the simplest on-off amplifiers working with pulse-width modulation. In such situations, at a constant power supply amplitude, the pulse width is a variable IOSSCH measurement parameter. Two examples illustrating the measurement of the IOSSCH together with the display of the immediately measured and filtered output signals $y_{m}$ and *y* are shown in Figure 2. They document the high time instability of mechatronic devices, which significantly depend on the performed maintenance (bearing lubrication). This instability is one of the motives for the introduction of automatic control and points to the need for a robust control design, which should ensure the minimum required level of control performance independent of such maintenance. As the human factor is also an important element of the design of automatic control, one of the most important goals of the INDUSTRY 4.0 initiative [49,50] is the digitization and automation of the overall control design.

### 2.3. Approximation of Stable Step Responses According to Ziegler and Nichols

One of the most commonly cited methods for approximating stable step responses, inspired by Ziegler and Nichols [3], can be summarized in the following steps.

First, set an allowable initial value ($u_{1}$) of the control variable (plant input) and hold it constant until the transients decay. Measure the corresponding (filtered) value of the output $y_{f1}$. If the filtered value is still noisy, calculate $y_{f1}$ by averaging several last measurements. Then reset the time $t_{0}=0$ and change the plant input from the value $u_{1}$ to the new value $u_{2}$ and record the filtered output (graphically or as a series of samples in the computer’s memory) until the transients decay (until $t=t_{max}$). Measure the new steady-state value of the output $y_{f2}$ and calculate the static gain of the system *K*

(4) $$K=\frac{y_{f2}-y_{f1}}{u_{2}-u_{1}}$$

**Figure 3:**
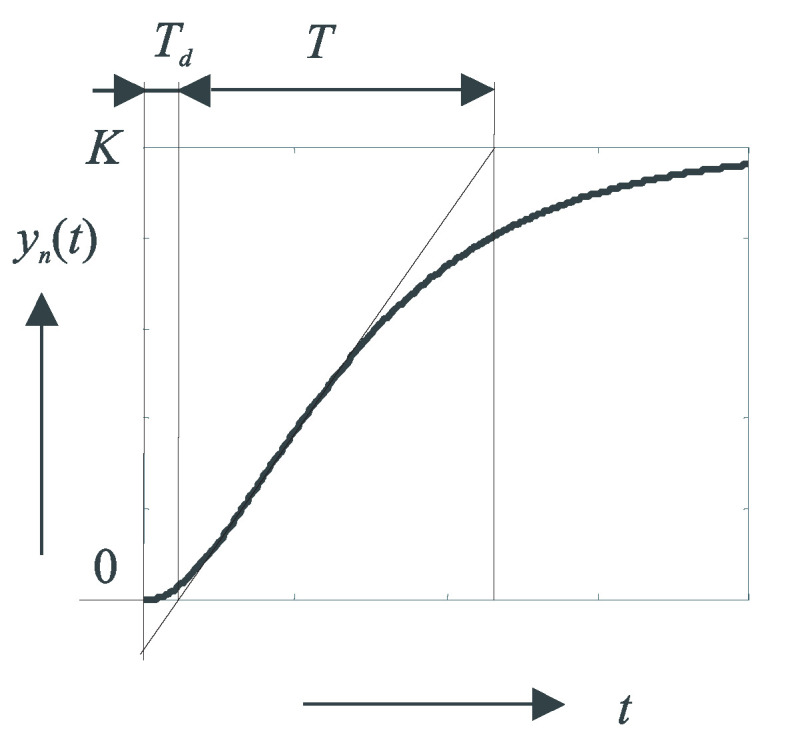
Approximation of a normalized stable step response based on a tangent drawn through the inflation point according to Ziegler and Nichols [3].

For a chosen input step $\Delta u=u_{2}-u_{1}$, calculate the normalized setpoint response $y_{n}\left(t\right)$ from $y_{f}\left(t\right)$ with time $t\in [0,t_{max}]$ (Figure 3), considering y(t)=0 for t≤0:

(5) $$y_{n}\left(t\right)=\Delta y_{f}/\Delta u;\;\Delta y_{f}\left(t\right)=y_{f}\left(t\right)-y_{f}\left(0\right);\;t\in \left[0,t_{max}\right]$$

From the parameters obtained by drawing the tangent line at the inflection point of $y_{n}\left(t\right)$, one can immediately compute two different linear models of the system. In the conventional linear model, which is locally approximated by the first-order transfer functions with time delay (FOTD)

(6) $$S_{a}\left(s\right)=\frac{\Delta Y(s)}{\Delta U(s)}=\frac{Ke^{-T_{d}s}}{Ts+1}=\frac{K_{s}e^{-T_{d}s}}{s+a};\;$$

the three parameters obtained are the transport delay $T_{d}$, the time constant *T* and the static gain of the system *K*. For a≠0, the model (6) can also be characterized by

(7) $$K_{s}=K/T;\;a=1/T.$$

However, it is possible to construct an even simpler “ultralocal” integrating process model with parameters $K_{s}$ (which gives the slope of the linearly increasing response given by the tangent line applied to the inflection point) and the dead time $T_{d}$

(8) $$S_{0}\left(s\right)=\frac{\Delta Y(s)}{\Delta U(s)}=\frac{K_{s}e^{-T_{d}s}}{s};\;$$

Here, the third parameter T=1/a→∞, which corresponds to a→0, cannot be evaluated at all.

**Remark** **1** (Different types of standardized setpoint responses). *If we want to use model (6), we must compute the static gain K and therefore wait until the transients decay. In contrast, model (8) can be obtained without determining the static gain, so the experiment can be shorter.*

**Remark** **2** (Local and ultralocal linear models). *The term “ultralocal” model is used in papers dealing with the design of model-free control using integral models based on the flatness theory elaborated by M. Fliess and colleagues. In previous work [51,52], the two linear (i.e., local) models (6) and (6) were denoted by the indices 1 or 0 depending on the number of terms in the Taylor series of the considered feedback nonlinearity.*

**Figure 4:**
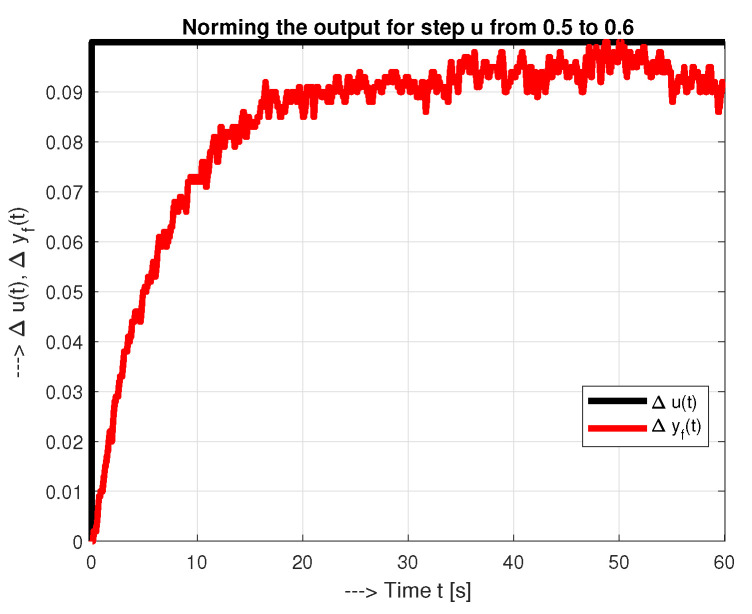
Norming one segment of the step response $y_{f}\left(t\right)$ corresponding to Figure 3 with the change of the input from u=0.5 to u=0.6.

- The fundamental weakness of both solutions with the model parameterization as shown in Figure 3 is the drawing of the tangent line through the inflection point. This was relatively easy to do with a traditional manual (graphical) solution. However, with numerical solutions, this is one of the most ill-conditioned tasks.
- The second weakness is the instability of the steady states in the system, which are sensitive to various disturbances from the environment. An example of such a step response is shown in Figure 4. The long measurement times can lead to large fluctuations in the process step response due to disturbances. The requirement for a steady-state measurement without disturbances can be a significant limitation in many applications.
- The third weakness is related to the fact that the obtained models (8) and (6) represent only linear, i.e., local models, which can no longer be sufficiently accurate when a larger range of operating points is applied due to the nonlinearity of the process. Therefore, it is meaningless to require “infinite” measurements to reach the steady state.

### 2.4. Local Identification of Local Linear Models

When we model a system, we must always keep in mind the purpose of the model. In our case, the purpose is to design controller that provides a tracking response of the process output that is as fast as possible, but at the same time sufficiently smooth, ideally without overshoot. From this point of view, the process model should have the highest accuracy in the mid to higher frequency range corresponding to the initial response of the process step response in the time domain. The time responses of models (6) and (8) when the step change is u(t)=1 are:

(9) $$y_{0}\left(t\right)=K_{s}\left(t-T_{d}\right),\;t>T_{d};\;\;y_{0}\left(t\right)=0,\;t\leq T_{d}$$

(10) $$y_{a}\left(t\right)=\frac{K_{s}}{a}\left(1-e^{-(t-T_{d})/T}\right),\;t>T_{d};\;\;y_{0}\left(t\right)=0,\;t\leq T_{d}$$

In the following time interval:

(11) $$t\in \left[0,t_{a}\right];\;T_{d}<t_{a}\leq t_{max}$$

the parameters of the models (9) and (10) are computed by searching over a grid of predefined values (defined, e.g., in Matlab as Ks=Ksmin:dK:Ksmax,Td=Tdmin:dT:Tdmax,a=amin:da:amax) such that the corresponding squares of the deviations

(12) $$S_{0}=\sum_{i=0}^{N}{\left(y_{ni}-y_{0i}\right)}^{2};\;S_{1}=\sum_{i=0}^{N}{\left(y_{ni}-y_{ai}\right)}^{2}$$

take the smallest possible values [13]. In (12), the values $y_{ni}=y_{n}\left(iT_{s}\right)$, $y_{0i}=y_{0}\left(iT_{s}\right)$ and $y_{ai}=y_{a}\left(iT_{s}\right)$ correspond to the measured $y_{n}$, $y_{0}$ and $y_{a}$ at the sampling interval $T_{s}$.

**Figure 5:**
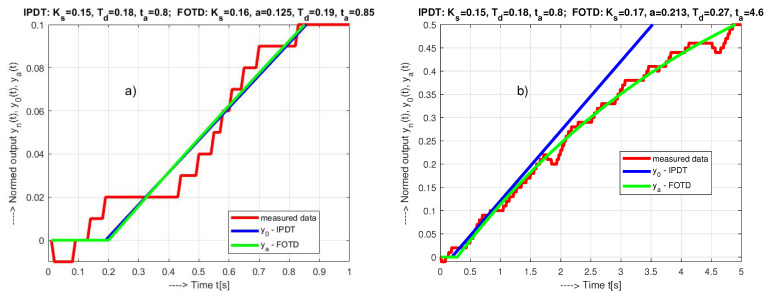
Approximations of an initial part of a normed step response y(t) for $t_{a}\leq 1$ (**a**) and $t_{a}\leq 5$ (**b**) with model parameters corresponding to the maximal values $T_{d}$ from particular “optimal” solutions fulfilling conditions (12).

Therefore, in addition to selecting the appropriate model parameters grid size and fragmentation, the time interval of the process response $t_{a}$ used for model identification should also be selected. It is obvious that $t_{a}>T_{d}$. No control will occur until $T_{d}$ expires, so you need to prepare for the worst-case scenario. Therefore, when searching for parameters for models (6) and (8) at different values of $t_{a}$, the models with the highest value of $T_{d}$ should be chosen. Such an approach takes into account that the value of $T_{d}$ plays a crucial role in tuning the controller parameters to achieve smooth transients without overshoot. For the process model (6) (based on $y_{a}\left(t\right)$ in the time domain), higher values of $t_{a}$ are generally expected to be used. However, if $t_{a}$ and the process noise are too large (as in the Figure 5b), regarding the accuracy of determining the fastest mode, the obtained model may not be accurate enough.

**Remark** **3** (Approximating the fastest mode). *Regarding the accuracy of determining the fastest mode, we can formulate a hypothesis that the approximation length $t_{a}$ should be chosen so that the optimal parameters $K_{s}$ and $T_{d}$ are similar for both $S_{a}\left(s\right)$ (6) and $S_{0}\left(s\right)$ (8).*

## 3. PI and PID Controller Tuning by the MRDP Method

### 3.1. 2DOF PI and PID Controllers

Although PID controllers are an industry standard, the possible implementation variants have not been sufficiently explored. There are at least two forms (structures) of PID controllers.

One of them is an ideal series PID controller:

(13) $$C\left(s\right)=\frac{U(s)}{E(s)}=K_{p}\frac{\left(1+sT_{i}\right)\left(1+T_{D}s\right)}{sT_{i}}=K_{p}\left(1+\frac{1}{T_{i}s}\right)\left(1+T_{D}s\right)$$

where $K_{p}$ is the controller gain, $T_{i}$ is the integral, and $T_{D}$ is the derivative time constant. The second form is an ideal parallel PID controller:

(14) $$C\left(s\right)=\frac{U(s)}{E(s)}=K_{p}\frac{1+sT_{i}\left(1+T_{D}s\right)}{sT_{i}}=K_{p}\left(1+\frac{1}{T_{i}s}+T_{D}s\right)$$

Please note that the parallel form is more general, since any series structure can be represented by the parallel structure, while the converse is not true. Please note that the optimal controller parameters for one structure are not optimal for the other (the parameters must be recalculated). As soon as we use both controllers at the same time, their parameters will need to be clearly distinguished by a suitable choice of symbols (see Section 3.4). The PI controllers are simplified PID controllers corresponding to $T_{D}=0$. The 2DOF PID or PI controllers represent an extension of a PID controller C(s) with one degree of freedom (1DOF) by a setpoint prefilter $F_{p}\left(s\right)$. The prefilter is added to speed up setpoint responses and avoid overshoot of the process output.

The anti-windup solution for the series PID controller is realized with the structure modeling pneumatic regulators, which were created before the Second World War with the original name “Pre-Act”. The integral action is accomplished by positive feedback from the controller output through the first-order filter with a time constant $T_{i}$ (see Figure 1 and note that $K_{d}=K_{p}T_{D}$). If the limitations of the control action are neglected, we obtain a combination of PD controller $K_{p}\left(1+T_{D}s\right)$ and positive feedback with a delay of $1/(1+T_{i}s)$, yielding the transfer function (13)

(15) $$C\left(s\right)=\frac{U(s)}{E(s)}=\frac{K_{p}\left(1+T_{D}s\right)}{1-1/\left(1+sT_{i}\right)}=K_{p}\frac{\left(1+T_{i}s\right)\left(1+T_{D}s\right)}{T_{i}s}$$

In the analysis of anti-windup controllers (see e.g., [53,54]), it is necessary to distinguish two modes of behavior—“control” mode, when the limitation is not active and the dynamics is determined by the transfer function C(s), and “tracking” mode, which is activated after reaching saturation limits, when the controller state is tracked so that it matches given inputs and outputs. With the scheme used, the tracking time constant $T_{t}=T_{i}$, which according to [53] represents an unnecessarily large value, leading to the output overshooting. However, the overshooting can be avoided if, in the factorization of the numerator C(s) discussed in Section 3.4, we choose as $T_{i}$ the smaller one of two possible values.

### 3.2. Optimal PI Tuning by TRDP Method

The goal of the Multiple Real Dominant Poles (MRDP) method is to avoid slow dominant closed-loop poles by choosing equal multiple closed-loop poles (see, e.g., [23,55,56,57] and references therein). The existence of an MRDP solution must be supported by a suitable controller structure and a sufficient number of equations to determine the controller parameters. If there are several possible solutions with multiple dominant poles, the most stable solution is chosen, i.e., the solution that lies in the left complex half-plane closest to the imaginary axis. The transfer function of the closed loop using the PI controller and the FOTD system (6) is

(16) $$\begin{array}{c} F_{cl}\left(s\right)=\frac{C(s)S(s)}{1+C(s)S(s)}=\frac{K_{p}K_{s}\left(1+T_{i}s\right)}{T_{i}s\left(s+a\right)e^{T_{d}s}+K_{p}K_{s}\left(T_{i}s+1\right)} \end{array}$$

The “optimal” controller tuning must achieve a triple real dominant pole (TRDP) of the characteristic quasi-polynomial:

(17) $$P\left(s\right)=T_{i}s\left(s+a\right)e^{T_{d}s}+K_{p}K_{s}\left(T_{i}s+1\right)$$

when it is possible to write $P\left(s\right)={\left(s-s_{o}\right)}^{3}P_{0}\left(s\right)$, by satisfying the conditions

(18) $$P\left(s_{o}\right)=0,\;\dot{P}\left(s_{o}\right)=0,\;\ddot{P}\left(s_{o}\right)=0$$

The optimal controller parameters, denoted by the subscript *o*, can be calculated as follows [30]

(19) $$\begin{array}{c} s_{o}=-\frac{A_{d}+4-S_{d}}{2T_{d}}\;,\;\;A_{d}=aT_{d}\;,\;\;S_{d}=\sqrt{A_{d}^{2}+8}\\ K_{po}=\frac{K_{o}}{K_{s}T_{d}};\;K_{o}=\left(S_{d}-2\right)e^{(S_{d}-A_{d}-4)/2}\\ T_{io}=\tau_{i}T_{d};\;\tau_{i}=\frac{2(2-S_{d})}{A_{d}^{2}+2A_{d}+28-\left(A_{d}+10\right)S_{d}} \end{array}$$

Denominator of an optimal prefilter

(20) $$F_{p}\left(s\right)=\frac{1+bs}{1+T_{i}s}$$

cancels the zero of the closed-loop transfer function (16). Then, the transients of the closed loop can be sped up by cancelling one of the optimal dominant poles ($s_{o}$). This results in its optimum tuning $b_{o}$ as

(21) $$b_{o}=-\frac{1}{s_{o}}=\frac{2T_{d}}{A_{d}+4-S_{d}}$$

For the IPDT model (8), much simpler tuning formulas can be derived since a=0 [55,58] and

(22) $$\begin{array}{c} s_{o}=-\left(2-\sqrt{2}\right)/T_{d}\approx 0.586/T_{d};\\ K_{o}=2\left(\sqrt{2}-1\right)e^{\sqrt{2}-2}\approx 0.461;\;\tau_{i}=\left(2\sqrt{2}+3\right)\approx 5.828;\;b_{o}=\frac{T_{d}}{2-\sqrt{2}}\approx 1.707T_{d} \end{array}$$

### 3.3. Calculation of the Optimal Parallel PID Parameters

The optimal parallel PID setting for a FOTD system, which results in a quadruple real dominant pole (QRDP) in the closed-loop system, is described in [31]. Considering the closed-loop system

(23) $$\begin{array}{c} F_{cl}\left(s\right)=\frac{C(s)S(s)}{1+C(s)S(s)}=\frac{K_{p}K_{s}\left(1+T_{i}s\left(1+T_{D}s\right)\right)}{T_{i}s\left(s+a\right)e^{T_{d}s}+K_{p}K_{s}\left(1+T_{i}s\left(1+T_{D}s\right)\right)} \end{array}$$

the characteristic quasi-polynomial is

(24) $$P\left(s\right)=T_{i}se^{T_{d}s}\left(s+a\right)+K_{p}K_{s}\left(T_{i}T_{D}s^{2}+T_{i}s+1\right)$$

The “optimal” parameters $K_{po}$, $T_{Do}$ and $T_{io}$ satisfying the conditions $P\left(s_{o}\right)=0$, $\dot{P}\left(s_{o}\right)=0$, $\ddot{P}\left(s_{o}\right)=0$ and $\dddot{P}\left(s_{o}\right)=0$, are given as

(25) $$\begin{array}{c} s_{o}=-\frac{6+A-S}{2T_{d}}\;,\;\;A=aT_{d}\;,\;\;S=\sqrt{A^{2}+12}\\ K_{po}=\frac{K_{o}}{K_{s}T_{d}};\;K_{o}=\frac{S\left(A+12\right)-\left(A^{2}+2A+36\right)}{2}e^{(S-A-6)/2}\\ T_{Do}=\tau_{D}T_{d};\;\tau_{D}=\frac{S-2}{S\left(A+12\right)-\left(A^{2}+2A+36\right)}\\ K_{Do}=K_{po}T_{Do}=\frac{S-2}{2K_{s}}e^{(S-A-6)/2}\\ T_{io}=\tau_{i}T_{d};\;\tau_{i}=\frac{2(36+2A+A^{2}-\left(A+12\right)S)}{A^{3}+12A^{2}+36A+288-\left(A^{2}+12A+84\right)S} \end{array}$$

### 3.4. Calculation of Optimal Series PID Parameters

For the series PID controller, the optimal QRDP position $s_{o}$ is the same as for the parallel controller (25). To avoid tedious calculations and to highlight the main difference in tuning the parallel and series controllers, we obtain the parameters of the series PID controller from the parallel controller by equating the transfer functions C(s) of both controllers. By comparing the coefficients at equal powers of ‘s’ in (13) and (14) and using the indices “*s*” and “*p*”, we can derive the following relations.

(26) $$\begin{array}{c} K_{ps}T_{ip}=K_{pp}T_{is};\;T_{is}+T_{Ds}=T_{ip};\;T_{is}T_{Ds}=T_{ip}T_{Dp} \end{array}$$

Based on the solution of the last two equations with respect to $T_{is}$, the following is obtained

(27) $$\begin{array}{c} T_{is}=\left[T_{ip}\pm \sqrt{\left(T_{ip}^{2}-4T_{ip}T_{Dp}\right)}\right]/2\\ K_{ps}=K_{pp}\left[T_{ip}\pm \sqrt{\left(T_{ip}^{2}-4T_{ip}T_{Dp}\right)}\right]/\left(2T_{ip}\right)=K_{pp}\left[0.5\pm \sqrt{\left(0.25-T_{Dp}/T_{ip}\right)}\right]\\ T_{Ds}=T_{ip}\mp \sqrt{\left(T_{ip}^{2}-4T_{ip}T_{Dp}\right)}]/2 \end{array}$$

The expressions (27) show that there are two optimal sets of parameters for the series PID controller. We have already pointed out this ambiguity in [44], and the conclusion was that it has no practical impact when the system is linear. However, it turns out to be important for the design of an anti-windup PID structure with integral action generated by a positive feedback from the output of the constrained controller with time constant $T_{i}$, as shown in Figure 1 (all parameters of the controller correspond to the series PID). On the other hand, the optimal setting of the series PID controller is much simpler using the IPDT model (the subscript “s” is omitted for simplicity). The MRDP method yields for (8)

(28) $$\begin{array}{c} s_{o}=\left(\sqrt{3}-3\right)/T_{d};\;K_{o}=K_{po}K_{s}T_{d}=0.7239\\ \tau_{i}=T_{io}/T_{d}=3.4475;\;\tau_{D}=T_{Do}/T_{d}=0.284 \end{array}$$

Let us now consider an ideal series $C_{a}\left(s\right)$ controller

(29) $$\begin{array}{c} C_{a}\left(s\right)=K_{pa}\frac{\left(1+T_{a}s\right)\left(1+T_{b}s\right)}{T_{a}s}=\\ =K_{pa}\left(1+\frac{1}{T_{a}s}\right)\left(1+T_{b}s\right)=>T_{i}=T_{a};\;T_{D}=T_{b} \end{array}$$

with parameters $K_{p},T_{a},T_{b}$ satisfying the closed-loop quadruple real dominant pole condition, where $T_{a}$ has been interpreted as $T_{i}$ and $T_{b}$ as $T_{D}$. This controller is obviously equivalent to the controller

(30) $$\begin{array}{c} C_{b}\left(s\right)=K_{pb}\left(1+\frac{1}{T_{b}s}\right)\left(1+T_{a}s\right)\\ K_{pb}=K_{pa}\frac{T_{b}}{T_{a}};\;\;=>T_{i}=T_{b};\;\;T_{D}=T_{a} \end{array}$$

Although the first set of parameters (28) was chosen to yield values for $\tau_{i}$ and $K_{o}$ that are close to PI control (22), the coefficients in (30) yield the second alternative with significantly different parameters ($K_{o}=0.0596$). The importance of this ambiguity can be shown in the case of constrained control (with the controller output limitations).

### 3.5. Prefilter Calculation

When changing from a parallel to a series PID controller, it is also necessary to change the calculation of the optimal prefilter, which is assumed as follows

(31) $$F_{p}\left(s\right)=\frac{1+bs+cs^{2}}{\left(1+T_{i}s\right)\left(1+T_{D}s\right)}$$

In the simplest case, it is designed to compensate for the zeros of the closed-loop transfer function

(32) $$\begin{array}{c} F_{cl}\left(s\right)=\frac{C(s)S(s)}{1+C(s)S(s)}=\frac{K_{p}K_{s}\left(1+T_{i}s\right)\left(1+T_{D}s\right)}{T_{i}s\left(s+a\right)e^{T_{d}s}+K_{p}K_{s}\left(1+T_{i}s\right)\left(1+T_{D}s\right)} \end{array}$$

With the trivially chosen setpoint weights

(33) $$b_{0}=c_{0}=0$$

the entire control transfer function becomes an I-PD controller. In the general case, the prefilter parameters can be derived from the polynomial equations

(34) $$\begin{array}{c} N_{1}\left(s\right)=\left(1-s/s_{o}\right)=1+\frac{2T_{d}}{6+A-S}s\;,\;\;A=aT_{d}\;,\;\;S=\sqrt{A^{2}+12}\\ N_{2}\left(s\right)={\left(1-s/s_{o}\right)}^{2}={\left(1+\frac{2T_{d}}{6+a-S}s\right)}^{2} \end{array}$$

to cancel out one or two dominant poles $s_{o}$. For (8) the result is

(35) $$\begin{array}{c} b_{1}=0.7887T_{d};\;c_{1}=0;\\ b_{2}=1.5774T_{d};\;c_{2}=0.6220T_{d}^{2}. \end{array}$$

In the experimental evaluation, we preferred to maintain the continuity of the course of the reference signal and so we worked only with c=0.

## 4. Time and Shape-Related Performance Measures

The integral of absolute error (IAE) is often used to evaluate the speed of closed-loop response:

(36) $$IAE=\int_{0}^{\infty }\left|e(t)\right|dt\;\;;\;\;\;e=w-y\;\;.$$

Since the controller optimization based on the IAE criterion leads to overshoot of the output in most cases, additional constraints must be applied to achieve a monotonic response. In this work, the additional optimization constraints are considered in the form of shape-related constraints [2,14]. These are based on the concept of monotonicity, where the monotonicity measure is proposed as

(37) $$TV_{0}\left(y\right)=\sum_{i=0}^{\infty }\left|y_{i+1}-y_{i}\right|-\left|y_{\infty }-y_{0}\right|$$

i.e., as the difference between the total signal path (*y*) (sum of increments $\left|y_{i+1}-y_{i}\right|$) and the minimum possible path required to change the signal from the initial state ($y_{0}$) to the final state ($y_{\infty }$). For monotonic responses (with no overshoot or undershoot of y(t)), $TV_{0}\left(y\right)=0$. Otherwise, $TV_{0}\left(y\right)>0$.

The evaluation of signal shapes based on deviations from monotonicity can be viewed as a generalization of the evaluation approaches used in the early stages of automatic control in relay time-optimal control. In this context, references to Feldbaum’s theorem [14,15,59,60] can be found in the older literature. According to this theorem, to move the output of an *n*th-order system (with full relative order) from one steady state to another, *n* control intervals are required in which a controlled variable changes its values from one limit to the opposite. Such a rectangular pulse can be considered to be a limiting case of two monotonically increasing/decreasing responses separated by an interval with a constant value of the constraint. For example, a simple integrator requires an input signal consisting of two monotonic intervals for a monotonic output transition of the steady state [58].

To evaluate deviations from such an ideal single-pulse waveform (1P) [58] consisting of two monotonic intervals separated by an extreme value $u_{m}\notin \left(u_{0},u_{\infty }\right)$ that lies outside the interval of the initial and final output values $u_{0}$ and $u_{\infty }$, the measure $TV_{1}\left(u\right)$

(38) $$TV_{1}\left(u\right)=\sum_{i}\left|u_{i+1}-u_{i}\right|-\left|2u_{m}-y_{\infty }-u_{0}\right|$$

can be used. To separate the measured deviations due to the change of the steady state from the deviations caused by the process noise, the evaluation interval can be restricted to the time at which the process response practically settles ($\left|e\left(t\right)\right|\leq e_{0}$, where $e_{0}$ is chosen according to the noise level). Simpler relationships for the IPDT model can be used to demonstrate the effect of $T_{d}$ on the rate of transients. For example, in the PID control yielding ideal shapes (with zero deviations at the plant output), the optimal IAE values characterizing unit setpoint step responses can be calculated using the Laplace transform [44] as integral of error (IE) values, when

(39) $$IAE=T_{i}+T_{D}-b$$

With the simplest prefilters (33) and (35)

(40) $$\begin{array}{c} IAE_{0}=3.732T_{d};\;IAE_{1}=2.9433T_{d};\;IAE_{2}=2.1546T_{d} \end{array}$$

The results of the analysis of the influence of $T_{d}$ on the speed of transients in the design using MRDP method make it possible to formulate the following note.

**Remark** **4** (Motivation to search for models with a maximum dead-time value). *If we used a smaller $T_{d}$ than the actual one when setting the controller, the transients would be accelerated. However, since the TRDP corresponds to a limit setting at which oscillations do not yet occur, this would be at the cost of disrupting the ideal waveforms. To avoid this, we look for the IPDT model with the largest $T_{d}$ value when identifying the plant.*

## 5. Illustrative Example

**Figure 6:**
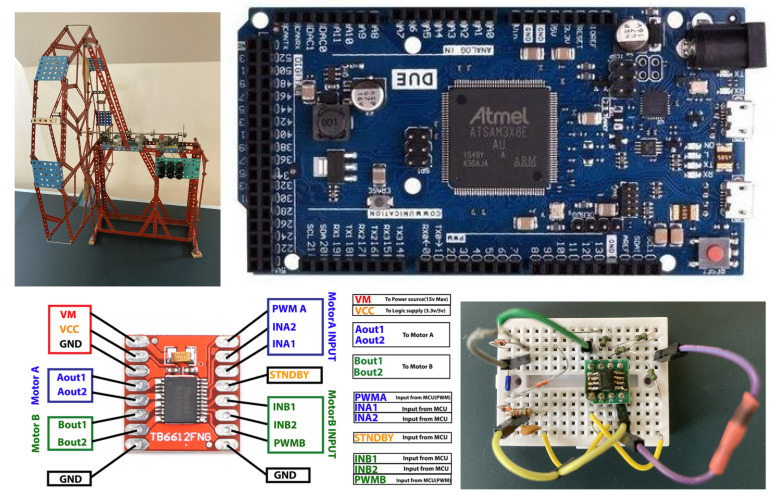
Components of the considered electromechanical Arduino-Due-based speed system.

Mechatronics is an initiative started in Japan around 1970 that aims to improve the functionality of various products by making them more accurate, flexible, adaptable, or user-friendly - all with the goal of reducing production costs. With similar goals in mind and to test the dynamics of electric vehicle models, in 1982 we built a low-cost speed control system with three commutator DC motors. The mechanical construction of the vehicle model was made using the Meccano kit from Merkur (Figure 6). At that time, the device was controlled by a HP-85 computer, which allowed the use of self-adjusting and adaptive controllers. The sampling time was between 0.1 and 0.3s, which is reasonable given the main time constant of the system (about 5 s).

From a mechanical point of view, the car model was not very close to the actual car, but it was suitable for testing and improving the self-adjusting and adaptive control algorithms mentioned above, including the study of nonlinear and constrained systems, time-varying systems, measurement and quantization noise, resonances, etc., which were later applied to various systems. The advent of embedded computers with much higher computational power in recent decades enabled the experimentation of various control approaches, which were previously not possible due to the limited computational power of microprocessors. Here, the aforementioned electromechanical system proved to be a suitable candidate to demonstrate recent advances in control approaches and to show that many control shortcomings of the past can be easily addressed today.

The device consists of a DC motor driven by a pulse-width modulated (PWM) motor driver that converts the process input voltage into 7V high pulses of the same frequency and varying duty cycle. The displayed process input value *u* is normalized between 0 and 1, where 1 means a duty cycle of 100% or constant 7 V and 10 ms at the motors. The process output is the rotational speed of the system measured by the third DC motor. To attenuate the significant measurement noise (see Figure 2), highlighted due to the use of a simple motor instead of a special tachodynamo, the 2nd order filter in Figure 1 was extended to the 4th order binomial filter (1) used with time constant $T_{f}=50$ ms at sampling period $T_{s}=0.1$ ms, which satisfies condition (2). It should be borne in mind here that the sampling period $T_{s}$ cannot be reduced arbitrarily, even if the signal processing speed would allow it. As $T_{s}$ decreases, also useful output changes per sampling period decrease, increasing the effect of quantization noise. At the selected $T_{s}$ value, the effect of output level quantization against measurement noise was still negligible. The sampling period used for the control algorithm was $T_{s}=10$ ms. For all measurements, the output speed of the process is normalized to the fastest speed, which is 13.7 revolutions per second (RPS). Therefore, the indicated speed in the diagrams is between 0 and 1. Experiments with the built device complement similar research carried out with a speed system consisting of a DC motor with an incremental speed sensor [61]. Thanks to very simple construction enabling the work with a higher moment of inertia and a larger mechanical time constant (and an easy verification of the presented research by the readers), the sampling period can be chosen in a wide range. With the chosen experiment setup, we do not want to question the advantages of incremental sensors, but to explore the possibilities of improving the noisy output from analog sensors, which are still important in a high number of practical applications. The sensing of the speed by the commutator tachodynamo leads to the examination of a substantially different situation with a different character of the acting noises. The source of the signal fluctuation is mainly the rotation of the rotor winding in a variable magnetic flux and the switching of individual coils. By shortening the sampling period and increasing the inertia of the filters, we obtain a roughly constant speed signal from the measured signal at a constant rotational speed. The experimental results fully confirm these expectations.

When evaluating the individual step responses in Figure 2, it is clear that the system is nonlinear and time-varying: After prolonged experimentation, the motor characteristics change with time due to the increased motor temperature. They also change significantly after each lubrication of the bearings. The variations in velocity in the vicinity of steady states are mainly due to imperfect mechanics and resonances that occur. This means that the steady-state identification may not be accurate enough and repeatable to achieve a reliable design of the desired closed-loop dynamics. Therefore, the classical identification methods introduced by Ziegler and Nichols [3] should be modified.

### 5.1. Specifying the Local and Ultralocal Linear Process Approximations

The analysis and modifications of Ziegler and Nichols’ method have been a constant since the 1940s. Let us mention here at least the modifications concerning the digitization of the model transfer function [12]. Here we will further develop the model specifications according to [13]. However, the actual list may be more extensive as new approaches are developed regularly. The basic motivation for the proposed modifications is to increase the reliability in real life (applications) and to obtain more efficient closed-loop transients. We add to them the need for the widest possible digitization of the overall design. To achieve this, the new methods of controller design and the new criteria for evaluating the performance of transients are used. However, the fundamental problem of these methods is the reliable determination of the model parameters (in our case (6) and (8)). The design of a controller can be concise only if the model used in its design approximates with sufficient accuracy the fastest components of the transients, which corresponds to an accurate estimation of the process in the higher frequency range. If we accurately approximate slow components with it (i.e., if we focus on estimation of the process model at low frequencies), the design can be completely degraded by the behavior of fast components. The fact that a model used excellently approximates the measured process reaction curve over a wide time, can be irrelevant for a fast and smooth setpoint tracking.

The models in Figure 5a were obtained by searching for optimal parameters on the grid defined by the intervals:

(41) $$\begin{array}{c} Td\in \left[0,50T_{s}\right],\;\Delta T_{d}=T_{s};\\ K_{s}\in \left[0.1,1.5\right],\;\Delta K_{s}=0.01;\\ T\in [2,8],\;\Delta T=0.1. \end{array}$$

In the presence of measurement noise, the basic way to refine the model parameters is to extend the evaluated interval. However, this does not apply indefinitely. For example, consider an identification interval specified by $t\in [0,t_{a}]$. Since optimal results cannot be expected for every identification interval, consider several approximations with $t_{a}\in \left[0.5,1\right]$, all of which have specific parameter values (41). The optimal setting in each case must satisfy conditions (12). The global optimum can be sought under the condition of the maximum value of $T_{d}$, since the dead time is usually the most limiting parameter of the controller design. Figure 5a shows that for $t_{a}\leq 1$, the responses of both models are practically the same. Therefore, the values of $K_{s}$ and $T_{d}$ of the two transfer functions (6)–(8) considered are approximately the same:

(42) $$\begin{array}{c} \mathrm{IPDT},\;t_{a}\leq 1:\;K_{s}=0.15;\;T_{d}=0.18;\;a=0;\;t_{ao}=0.8\\ \mathrm{FOTD}1,\;t_{a}\leq 1:\;K_{s}=0.16;\;T_{d}=0.19;\;a=0.125;\;T=1/a=8;\;t_{ao}=0.85 \end{array}$$

Here $t_{ao}$ denotes the identification interval that yields the optimal parameter set. If we extend the possible length of the identification to $t_{a}\in \left[0.5,5\right]$, we obtain

(43) $$\begin{array}{c} \mathrm{IPDT},\;t_{a}\leq 5:\;K_{s}=0.15;\;T_{d}=0.18;\;a=0;\;t_{ao}=0.8\\ \mathrm{FOTD}5,\;t_{a}\leq 5:\;K_{s}=0.17;\;T_{d}=0.27;\;a=0.213;\;T=1/a=4.7;\;t_{ao}=4.6 \end{array}$$

The IPDT model has not changed, but the FOTD model, which estimates the process response more accurately, leads to significantly larger values of all determining parameters than for $t_{a}\in \left[0.5,1\right]$.

### 5.2. Experimenting with PI Control

By choosing the identification interval $t_{a}\leq 1$ the parameters of the PI controllers (19)–(22) are

(44) $$\begin{array}{c} PI_{0}:\;K_{Po}=17.07995526;\;T_{io}=1.049116873;\;b_{o}=0.3072792204\\ PI_{a1}:\;K_{Po}=14.99317409;\;T_{io}=1.034359438;\;b_{o}=0.3179322586 \end{array}$$

Comparing the controller parameters obtained from both models, it can be seen that the biggest difference is in the gains $K_{Po}$. Repeating the identification in a larger interval $t_{a}\leq 5$, the parameters corresponding to FOTD5 are as follows

(45) $$\begin{array}{c} PI_{a5}:\;K_{Po}=9.771989345;\;T_{io}=1.338369226;\;b_{o}=0.4395610608 \end{array}$$

In this case, the controller parameters obtained from the IPDT and FOTD models differ significantly.

**Figure 7:**
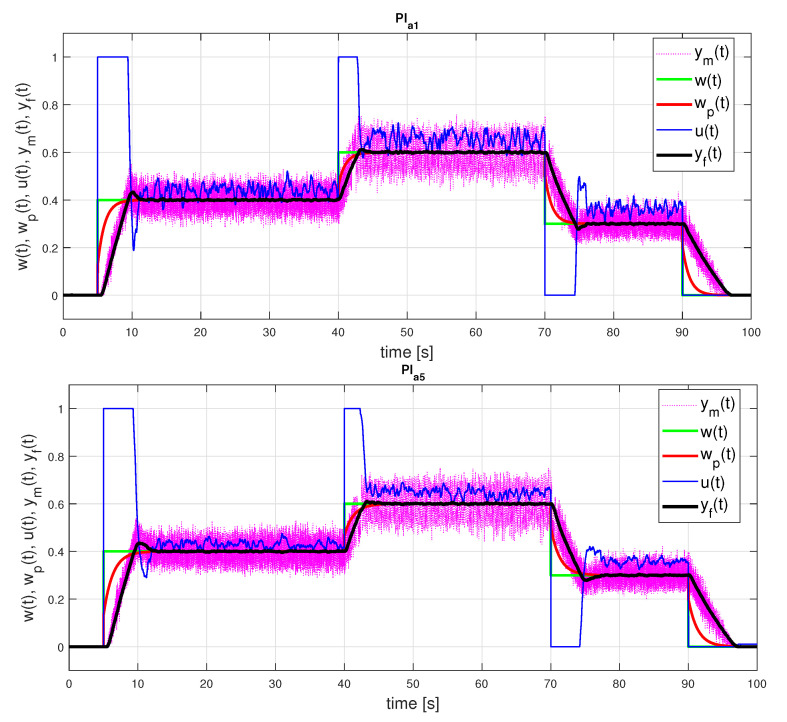
PI control: setpoint responses corresponding to FOTD approximations of an initial part of a normed step response $y_{n}\left(t\right)$ for $t_{a}\leq 1$ (42) with the parameters (44) (above) and for $t_{a}\leq 5$ (43) with the parameters (45) (below).

**Figure 8:**
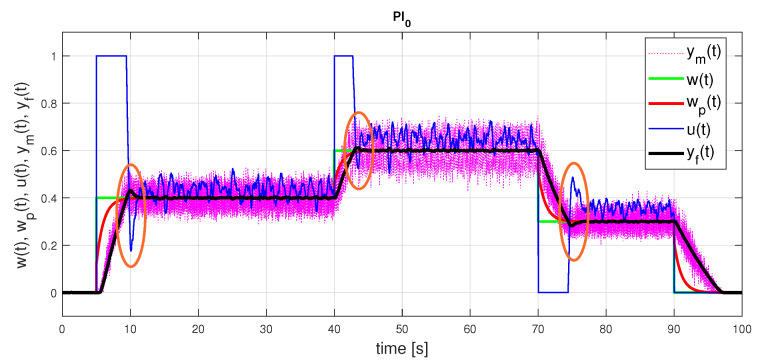
$PI_{0}$ control: setpoint responses corresponding to IPDT approximations of an initial part of a normed step response $y_{n}\left(t\right)$ (42) with the parameters (44) and with emphasis on areas including overshooting output requiring compensation by an additional input pulse.

Figure 7 and Figure 8 show the setpoint responses of the PI controllers designed according to the two considered models and identification intervals. A slight overshoot of the output responses corresponding to the FOTD model with $t_{a}\leq 1$ is a consequence of the limitation of the control signal and the nonlinearity of the process. Indeed, the overshoot does not occur for smaller setpoint changes. However, it should be noted that the overshoot would be much larger without anti-windup protection.

In the case of the FOTD model over a longer identification interval $t_{a}\leq 5$, a lower controller gain leads to lower $TV_{1}\left(u\right)$ values (see Table 1). A small overshoot of the output occurs in both process models used. It is obvious that neither the transients nor the performance measures are better when a more complex FOTD model is used (for both $t_{a}\leq 1$ and $t_{a}\leq 5$).

### 5.3. Experimentation with PID Control

The calculated PID control parameters (19)–(22) for $t_{a}\leq 1$ are

(46) $$\begin{array}{c} PID_{10}:\;\\ K_{Po}=26.80948841;\;T_{io}=0.6205422427;\;T_{Do}=0.05122690297;\;b_{o}=0.1419615242\\ PID_{20}:\;\\ K_{Po}=2.213172556;\;T_{io}=0.05122690297;\;T_{Do}=0.6205422427;\;b_{o}=0.1419615242\\ PID_{1a1}:\;\\ K_{Po}=23.61125885;\;T_{io}=0.6289503085;\;T_{Do}=0.05389188106;\;b_{o}=0.1484626127\\ PID_{2a1}:\;\\ K_{Po}=2.023140996;\;T_{io}=0.05389188106;\;T_{Do}=0.6289503085;\;b_{o}=0.1484626127 \end{array}$$

**Figure 9:**
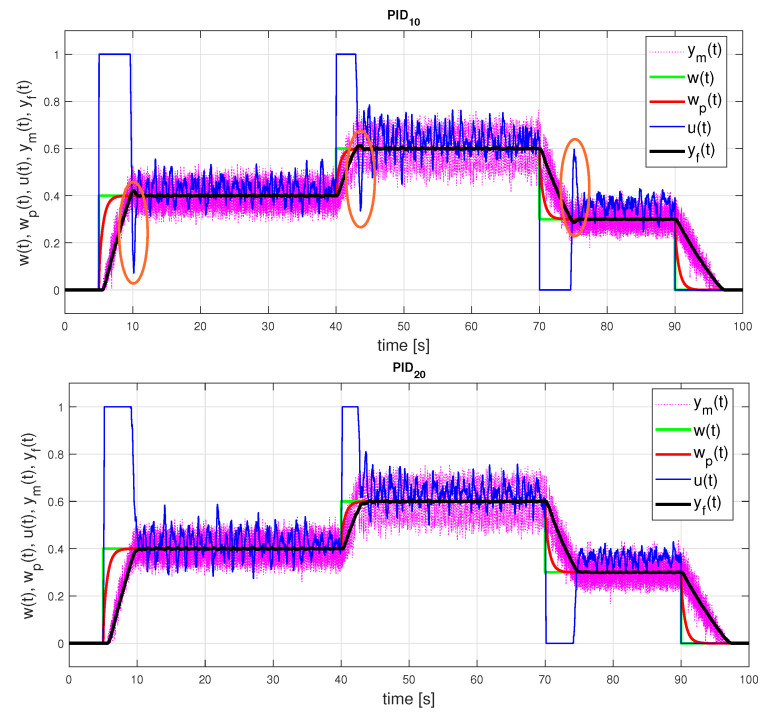
PID control: setpoint responses corresponding to IPDT approximation of an initial part of a normed step response $y_{n}\left(t\right)$ for both possible sets of the controller parameters (46) with emphasis on areas with overshooting output and with excessive input pulse of $PID_{10}$.

The largest differences in the controller parameters exist for the gain $K_{Po}$ in both models. Performance measures of the responses corresponding to the FOTD model in Table 1 show that despite the large differences in the controller parameters, the differences in the closed-loop responses are relatively small. The responses corresponding to the IPDT model in Figure 9 are very close to the responses obtained with a more complex FOTD model. However, a closer look at the responses reveals that we obtain monotonic (and nearly time-optimal) process output responses and 1P control signal responses only for the second set of controller parameters with a smaller value of $K_{p}$. The control loop responses corresponding to the first set of parameters exhibit small overshoots of the process output responses (increased PO values in Table 1 and slightly increased $TV_{0}\left(y\right)$ values) and the second impulse on the controller output response.

By evaluating the controller parameter for $t_{a}\leq 5$ we obtain

(47) $$\begin{array}{c} PID_{1a5}:\;\\ K_{Po}=13.24788546;\;T_{io}=1.408562174;\;T_{Do}=0.07048046952;\;b_{o}=0.1484626127\\ PID_{2a5}:\;\\ K_{Po}=0.6628867394;\;T_{io}=0.07048046952;\;T_{Do}=1.408562174;\;b_{o}=0.1484626127 \end{array}$$

In this case, the difference of the controller parameters, except for the setpoint weighting factor *b*, is again obvious.

**Figure 10:**
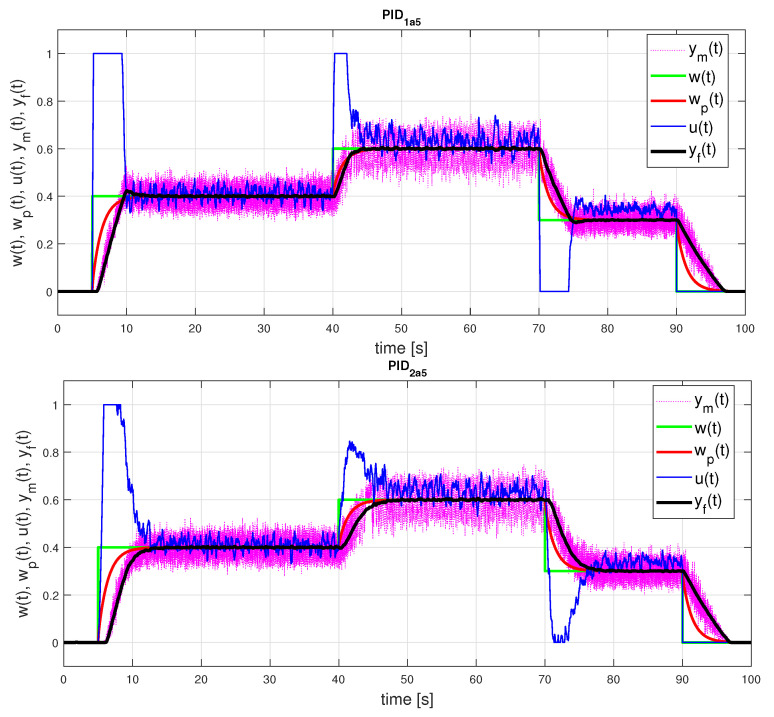
PID control: setpoint responses corresponding to FOTD approximations of a longer initial part of a normed step response y(t) for $t_{a}\leq 5$ with both possible sets of the controller parameters (47).

As can be seen in Figure 10, the differences in the control responses are relatively small despite the large differences in the controller parameters. The response of the $PID_{a52}$ controller is slightly less overshooting than that of $PID_{a51}$. The responses with $PID_{a52}$ are already slightly overdamped—the control signal does not go into saturation during the second and third setpoint changes. As a result, the IAE values increase.

## 6. Discussion

The performed experiments can be summarized in the groups of conclusions.

### 6.1. Step-Response-Based Plant Modeling

In Remark 4 we explained the motivation to look for approximations with a maximum value of $T_{d}$. We implemented the reasoning itself for the IPDT model, because for the FOTD model the corresponding relationships are too complex. In addition, the rate of transients depends on another parameter (*a*). However, the experimental results show that a different criterion will have to be sought to select the optimal FOTD model.

By analyzing the approximations at different $t_{a}$ (ta = 1: 0.1: 20) and choosing the maximum value of $T_{d}$, the optimal approximation IPDT (42) was found for $t_{a}=0.8$ and the optimal FOTD5 model (43) was obtained at $t_{a}=4.6$. Extending the approximation with the criterion formulated in this way no longer brings some improved results.

However, what do the FOTD approximations evaluated for higher values of $t_{a}$ bring? For example, the parameters of the FOTD20 model, obtained by least squares for $t_{a}=20$ and parameter grid (41) are

(48) $$\begin{array}{c} \mathrm{FOTD20}20,\;t_{a}=20:\;K_{s}=0.15;\;a=0.161;\;T=1/a=6.2;\;T_{d}=0.18 \end{array}$$

This model then yields parameters of the PI and PID control

(49) $$\begin{array}{c} PI_{a20}:\\ K_{Po}=16.84;\;T_{io}=0.965;\;b_{o}=0.311\\ PID_{1a20}:\\ K_{Po}=26.45;\;T_{io}=0.594;\;T_{Do}=0.051;\;b_{o}=0.148;\;c_{o}=0\\ PID_{2a20}:\\ K_{Po}=2.266;\;T_{io}=0.051;\;T_{Do}=0.594;\;b_{o}=0.148;\;c_{o}=0 \end{array}$$

The corresponding transients with PI control and both options of PID control in (see Table 1) just confirm the previously discussed properties: PI control and the first option of PID control show slight output overshooting and an additional control pulse at the input. Monotonic output changes correspond just to the 2nd set of controller parameters (49). However, when decreasing the steps in looking for the optimal least squares responses in the parameters $K_{s}$ and *T* (41) to one half and repeating the optimal model search with $t_{a}=20$ and $t_{a}=30$, we may see that with respect to FOTD20, the model FOTD20X changed significantly, whereas (considering the same number of decimal places) FOTD30X is nearly identical with FOTD20

(50) $$\begin{array}{c} \mathrm{FOTD}20X,\;t_{a}=20:\;K_{s}=0.145;\;a=0.155;\;T=1/a=6.45;\;T_{d}=0.1\\ \mathrm{FOTD}30X,\;t_{a}=30:\;K_{s}=0.150;\;a=0.161;\;T=1/a=6.20;\;T_{d}=0.18 \end{array}$$

**Figure 11:**
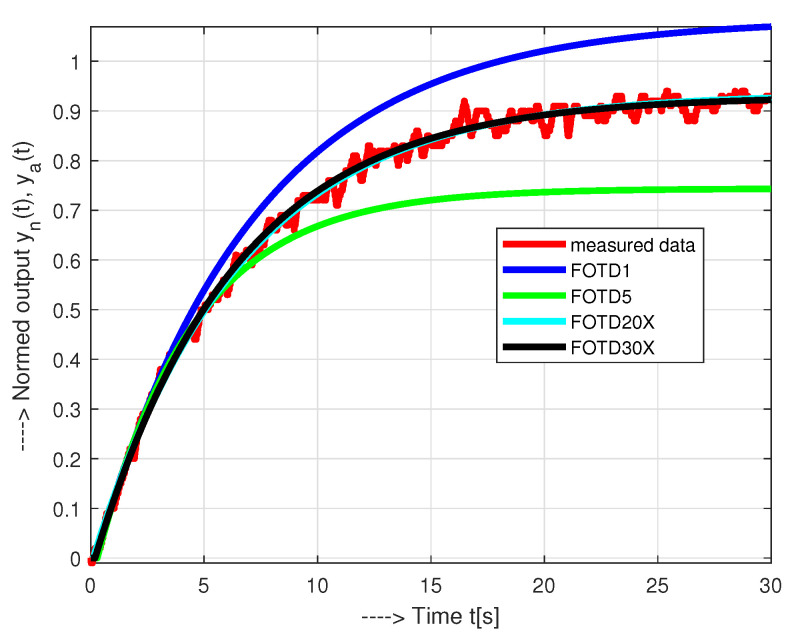
FOTD approximations of a normed step response $y_{n}\left(t\right)$ calculated for $t_{a}\leq 1$, $t_{a}\leq 5$, $t_{a}=20$ and $t_{a}=30$.

That is, the parameters *a* (or T=1/a) and $T_{d}$ may vary in a relatively broad range, whereas the parameter $K_{s}$ remains roughly constant. All the newly calculated values of $T_{d}$ are now smaller than $T_{d}=0.27$, obtained at $t_{a}=4.6$. Figure 11 shows that the time response of the FOTD1 and FOTD5 models resemble the actual time response of the process only in the first few seconds. Therefore, the approximation intervals $t_{a}\leq 1$, $t_{a}\leq 5$, $t_{a}=20$ or $t_{a}=30$ used are still short with respect to the total measurement time to steady state $t_{max}=60$ (in Figure 4), whereas the settling time is roughly 30 s. Responses corresponding to FOTD20X and FOTD30X are graphically difficult to distinguish, but this is no longer the case for *T* and $T_{d}$ values and even less for controller parameters.

On the one hand, this means that repeating the task for several values $t_{a}$, with matching the obtained values according to (12), loses its meaning for longer $t_{a}$. On the other hand, above results show that from a numerical point of view, the method of identifying FOTD model is poorly conditioned and should be avoided. If we extend the length of the approximation further, the more the effect of the various perturbations will become apparent, and we could perhaps reach completely unusable results and conclude that the Ziegler and Nichols method may not be practically applicable.

We may continue this analysis with evaluating parameters of the controllers corresponding to the obtained models FOTD20X and FOTD30X. For the PI controller we obtain parameters

(51) $$\begin{array}{c} PI_{a20X}:\;K_{Po}=31.56;\;T_{io}=0.557;\;b_{o}=0.168\\ PI_{a30X}:\;K_{Po}=16.84;\;T_{io}=0.966;\;b_{o}=0.300 \end{array}$$

**Figure 12:**
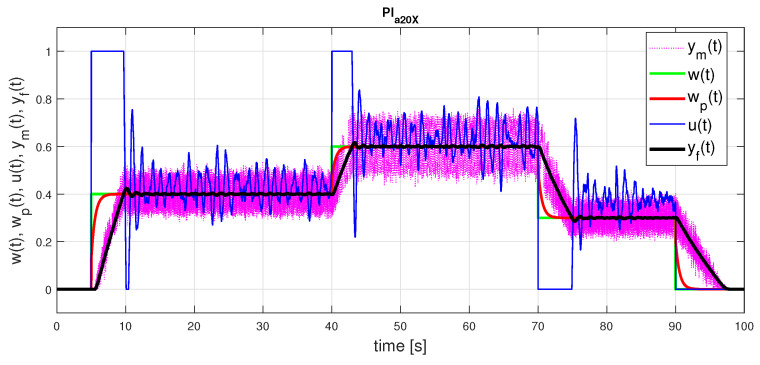
PI control: setpoint responses corresponding to FOTD20X approximation (50) achieved with $t_{a}=20$ for a modified grid of parameters (41) with two times finer steps for *T* and $K_{s}$ and the controller parameters (51).

If we mentioned above the high numerical sensitivity of determining the parameters of FOTD models, this is even more true for determining the parameters of PI controllers. In the case of graphically difficult-to-distinguish approximations of measured responses, we obtain almost double the differences in terms of determining the optimal controller gain. In addition, there are big differences in other parameters as well, which finally lead to much more oscillatory responses in Figure 12.

Even greater differences are shown in the case of the PID controller. Here we obtain

(52) $$\begin{array}{c} PID_{1a20X}:\\ K_{Po}=35.67;\;T_{io}=0.495;\;T_{Do}=0.011;\;b_{o}=0.078\\ PID_{2a20X}:\\ K_{Po}=0.782;\;T_{io}=0.011;\;T_{Do}=0.495;\;b_{o}=0.078\\ PID_{1a30X}:\\ K_{Po}=26.45;\;T_{io}=0.594;\;T_{Do}=0.051;\;b_{o}=0.140\\ PID_{2a30X}:\\ K_{Po}=2.266;\;T_{io}=0.051;\;T_{Do}=0.594;\;b_{o}=0.140 \end{array}$$

**Figure 13:**
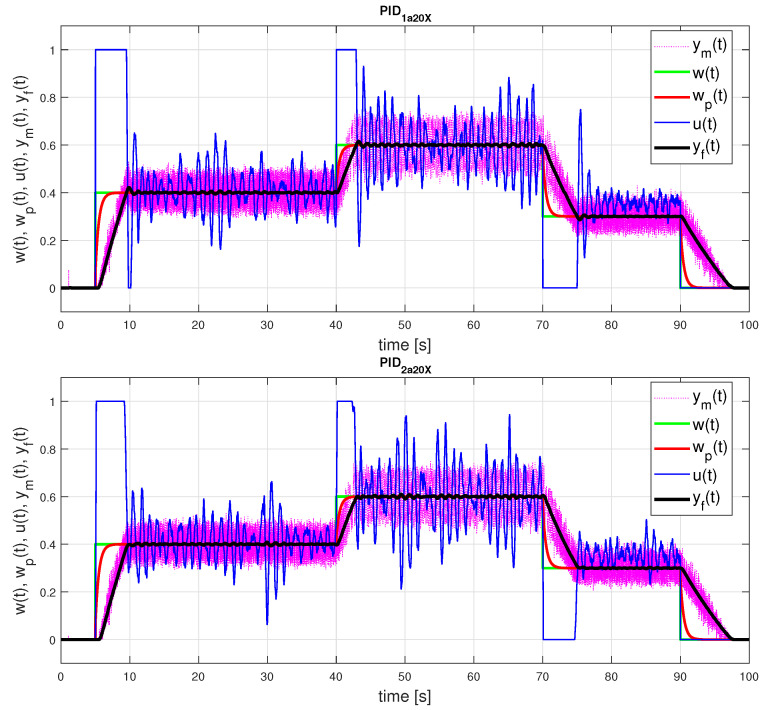
PID control: setpoint responses corresponding to FOTD20X approximation (50) achieved with $t_{a}=20$ for a modified grid of parameters (41) with two times finer steps for *T* and $K_{s}$ and the controller parameters (52).

Also these parameters lead to much more oscillatory responses in Figure 13.

So the basic question here is how do we recognize the most advantageous FOTD model? Surprisingly, the settings of both PI and PID controllers resulting from the FOTD20 and FOTD30X models are very close to the parameters calculated using the IPDT model. With the field of results of experimental verification, this points to the already mentioned principle (see Remark 3) that when determining the parameters of the FOTD model, we should consider their compliance with the parameters from the IPDT model as a criterion of their aptness, i.e., regarding the accuracy of determining the fastest mode, the optimal FOTD model has to be specified in such a way that its parameters $K_{s}$ and $T_{d}$ are similar for both $S_{a}\left(s\right)$ (6) and $S_{0}\left(s\right)$ (8) and thus yield also similar controller parameter values. Of course, when evaluating the suitability of the PID parameters (52) resulting from the FOTD20X model, another question arises as to what extent the selected sampling period $T_{s}=0.01$ s is sufficient for their implementation.

### 6.2. Two Options in PID Parametrization

Additionally, the paper comes to the conclusions that the use of a more complex PID controller can give faster transients with smaller output deviations from ideal shapes, even in the case of heavily noisy processes, than can be obtained with simpler PI controllers. This example is important regarding numerous applications in mechatronics, where great emphasis is placed on the fastest possible monotonic step responses without overshooting. When achieving such transients, control action constraints usually also apply. From this point of view, the use of anti-windup I-action accomplished in the form of feedback from the constrained output of the controller (as in Figure 2) proves to be interesting. It is realized with a delayed time constant $T_{i}$. However, when using a PI controller, output overshoot almost always appears. This can simply be avoided using a series PID controller but only with one of the two possible controller parameterizations. This fact was first pointed out in [44]. The realized experiments fully confirmed expectations from [44] and so they are definitely not a result of random parasitic effects and uncertainties arising under real-time control. Although it may seem surprising and counter-intuitive that a circuit that under linear control, gives the same transients for both possible sets of controller parameters, gives different properties in limiting the control action. It should be noted, however, that to examine the effect of a nonlinear saturation block (representing a special case of sector nonlinearity), the control loop must be transformed into an equivalent canonical circuit with saturation and a linear part [17,59,60,62]. In such a case, however, different controller parametrization corresponds to different transfer functions of the linear part, which may explain the different behavior. However, since the works in this area focus on stability, while not paying attention to the monotonicity of transients, given the limited scope of this article and its focus on experiments with the Arduino computer, we will avoid more detailed considerations here. The benefit of the new solution with PID control is even more valuable because traditional anti-windup controllers based on conditioning techniques [63] always lead to overshooting [2].

### 6.3. Final Recommendations

Finally, if we try to summarize the results of the carried-out experiments quantified by Table 1 into the final recommendations, regarding the emphasized advantages, they will be different:

If a nearly time-optimal transient responses (low IAE) with well-damped steady states (low $TV_{1}\left(u\right)$) are required, while overshooting up to 10% is not a problem, $PI_{a5}$ appears to be the most appropriate solution. $PI_{a1}$ and $PI_{0}$ offer roughly the same output at approximately twice the excessive input increments ($TV_{1}\left(u\right)$). The number of samples needed to determine the model has been reduced for $PI_{0}$, which makes it possible to shorten the identification of the system. The use of FOTD20, FOTD20X or FOTD30X models with a significantly longer identification phase and more demanding parameter evaluation does not bring any obvious advantages. Rather, it is accompanied by a problem which of the obtained models, which lead to a considerable variance of the controller parameters, is actually preferred. If it is necessary to reduce output overshooting and excessive output increments as much as possible, PID controllers corresponding to the second option parameters prove to be the most advantageous. For the upwards steps, $PID_{2a5}$ and $PID_{20}$ are most preferred, while when jumping downwards, $PID_{20}$ is the best. Again, the use of models with a longer approximation interval does not bring obvious advantages. If further smoothing of the responses is needed, it would be even more necessary to reduce the sampling period $T_{s}$.

## 7. Conclusions and Future Work

In the paper, some modifications to the oldest and most frequently cited method of setting the parameters of PI and PID controllers [3], resulting from the need to digitize them, have been proposed and tested. When trying to digitize the whole design, the first modification avoids the digital determination of the inflection point of the step response curve of the process. The proposed solution is not only numerically more stable, but also allows more accurate identification of the relevant process parameters. Instead of the original quarter-amplitude-damping, performance was evaluated based on deviations from ideal input and output responses formulated based on monotonicity. The optimal controller parameters are determined using the Multiple Real Dominant Poles method applied to PI and series PID controllers. The applied anti-windup protection ensures that the input and output responses of the process are close to the ideal shapes even for constrained systems. The study also showed that more complex FOTD process models offer little or no improvement over the IPDT models in the experiments provided. Moreover, the use of simple integrating process models, avoiding the identification of internal feedback parameters (and denoted therefore also as model-free control), is a cornerstone of modern control methods, such as advanced disturbance rejection control.

The third modification is the implementation of a low-pass filter on the measured process output. By reducing measurement noise, the filter improves the accuracy of process identification and significantly reduces the controller output noise during control. Therefore, the PID controller is also suitable for noisy processes. As already stated in some works [2,14], the control performance for noisy and uncertain processes can be further improved using controllers with higher-order derivatives.

## Figures and Tables

**Table 1:** Performance measures IAE, $TV_{0}\left(y\right)$ and $TV_{1}\left(u\right)$ corresponding to three steps for all experiments; PO—percentage overshoot; minimum—blue; maximum—red.

	Step from 0 to 0.4				Step from 0.4 to 0.6				Step from 0.6 to 0.3			
	IAE	${\mathrm{TV}}_{0}\left(y\right)$	${\mathrm{TV}}_{1}\left(u\right)$	**PO**	IAE	${\mathrm{TV}}_{0}\left(y\right)$	${\mathrm{TV}}_{1}\left(u\right)$	**PO**	IAE	${\mathrm{TV}}_{0}\left(y\right)$	${\mathrm{TV}}_{1}\left(u\right)$	**PO**
$PI_{0}$	**1.023**	0.096	1.154	8.00	0.314	**0.110**	1.678	6.00	**0.656**	**0.066**	0.870	6.00
$PI_{a1}$	1.043	0.106	1.158	8.25	0.312	0.094	1.296	6.00	0.667	**0.092**	1.096	**7.67**
$PI_{a5}$	1.064	0.098	**0.584**	**8.75**	**0.311**	0.088	**0.744**	**5.00**	0.679	0.072	**0.480**	7.33
$PI_{a20}$	**1.127**	**0.086**	0.918	8.00	0.316	**0.064**	0.972	6.00	0.710	0.074	0.874	6.33
$PI_{a20X}$	1.078	**0.118**	**3.000**	**6.00**	**0.324**	0.102	**3.060**	**8.00**	**0.729**	0.080	**2.298**	**4.67**
$PID_{10}$	1.056	0.080	2.530	5.50	**0.319**	0.126	4.204	6.50	0.693	0.066	2.062	4.67
$PID_{20}$	1.105	0.056	2.372	1.00	0.342	0.086	3.578	1.50	0.701	**0.032**	1.730	**0.33**
$PID_{1a1}$	1.061	**0.106**	2.920	**6.25**	0.326	0.106	3.464	5.50	0.681	0.054	1.922	4.33
$PID_{1a5}$	1.101	0.094	1.646	5.75	0.342	0.094	**2.476**	3.00	0.699	0.056	**1.140**	3.67
$PID_{2a1}$	1.084	0.078	2.740	1.25	0.334	0.116	3.974	4.00	**0.663**	0.054	2.026	1.00
$PID_{2a5}$	**1.342**	**0.032**	1.944	**0.25**	**0.583**	0.068	2.620	1.50	**0.886**	0.034	1.834	**0.33**
$PID_{1a20}$	1.143	0.072	2.066	5.25	0.322	0.076	2.758	6.00	0.703	0.068	2.456	**5.00**
$PID_{2a20}$	1.168	**0.032**	**1.628**	0.50	0.337	**0.044**	2.572	**1.00**	0.711	0.044	2.170	0.67
$PID_{1a20X}$	**1.012**	0.104	**3.026**	5.25	0.334	**0.160**	5.438	**7.50**	0.740	**0.078**	**2.468**	4.67
$PID_{2a20X}$	1.065	0.072	2.776	1.00	0.332	0.144	**5.592**	5.00	0.764	0.042	1.452	0.67

## Abbreviations

The following abbreviations are used in this manuscript:

1P	One Pulse, response with 2 monotonic segments (1 extreme point)
ADRC	Active Disturbance Rejection Control
ART	Average Residence Time
DC	Direct Current
IAE	Integral Absolute Error
IPDT	Integrator Plus Dead-Time
IOSSCH	Input-Output Steady-State Characteristic
MRDP	Multiple Real Dominant Pole
PID	Proportional-Integrative-Derivative
QRDP	Quadruple Real Dominant Pole
TV	Total Variation
TV${}_{0}$	Deviation from monotonicity (0P shape)
TV${}_{1}$	Deviation from 1P shape
TRDP	Triple Real Dominant Pole
